# Outcome of CRH stimulation test and overnight 8 mg dexamethasone suppression test in 469 patients with ACTH-dependent Cushing’s syndrome

**DOI:** 10.3389/fendo.2022.955945

**Published:** 2022-10-06

**Authors:** Mario Detomas, Katrin Ritzel, Isabella Nasi-Kordhishti, Stefan Wolfsberger, Marcus Quinkler, Marco Losa, Viola Tröger, Matthias Kroiss, Martin Fassnacht, Greisa Vila, Jürgen Bernd Honegger, Martin Reincke, Timo Deutschbein

**Affiliations:** ^1^ Department of Internal Medicine I, Division of Endocrinology and Diabetes, University Hospital, University of Würzburg, Würzburg, Germany; ^2^ Medizinische Klinik und Poliklinik IV, Klinikum der Universität München, Munich, Germany; ^3^ Department of Neurosurgery, University of Tübingen, Tübingen, Germany; ^4^ Department of Neurosurgery, Medical University of Vienna, Vienna, Austria; ^5^ Endocrinology in Charlottenburg, Berlin, Germany; ^6^ Department of Neurosurgery, Instituto Scientifico San Raffaele, University Vita-Salute, Milan, Italy; ^7^ Clinical Division of Endocrinology and Metabolism, Department of Internal Medicine III, Medical University of Vienna, Vienna, Austria; ^8^ Medicover Oldenburg MVZ, Oldenburg, Germany

**Keywords:** ACTH, Cushing's disease, Cushing’s syndrome, CRH stimulation test, diagnosis, ectopic, endogenous hypercortisolism, high dose dexamethasone suppression test

## Abstract

**Objective:**

To evaluate diagnostic accuracy of the corticotropin-releasing hormone (CRH) stimulation test and the overnight 8 mg dexamethasone suppression test (DST) for the differentiation of Cushing’s disease (CD) and ectopic Cushing’s syndrome (ECS).

**Methods:**

Retrospective study in 6 European centers. Inclusion criteria: patients with a) overt adrenocorticotropin (ACTH)-dependent Cushing’s syndrome at the time of dynamic testing, b) histopathological confirmed tumors and/or c) postoperative biochemical remission and/or adrenal insufficiency. Optimal cut-offs were calculated via receiver operating characteristic (ROC) analysis using CD as reference.

**Results:**

469 patients were analyzed [78% females; median age 43 years (IQR 19)]. CRH test and overnight 8 mg DST were performed in 420 [CD, n=394 (94%); ECS, n=26 (6%)] and 237 patients [228 CD (96%), 9 ECS (4%)]. Both tests were performed in 205 patients (44%). The post-CRH %-increase at 30 minutes of both ACTH (cut-off ≥31%, sensitivity 83%, specificity 85%, AUC 0.81) and cortisol (cut-off ≥12%, sensitivity 82%, specificity 89%, AUC 0.86) discriminated best between CD and ECS. A test duration of >60 minutes did not improve diagnostic performance of the CRH test. The optimal cortisol cut-off for the %-suppression during the 8 mg DST was ≥55% (sensitivity 80%, specificity 78%, AUC 0.75).

**Conclusion:**

The CRH test has equivalent sensitivity but higher specificity than the 8 mg DST and is therefore the test of first choice. The diagnostic outcome of ACTH and cortisol is well comparable, however, sampling beyond 60 minutes post-CRH does not provide diagnostic benefits.

## Introduction

Adrenocorticotropin (ACTH) dependent glucocorticoid excess is the most frequent cause of endogenous Cushing’s syndrome. The underlying ACTH source can be located either in the pituitary (so called Cushing´s disease, CD) or - less likely - extra-sellar, with most tumors being found in the lungs (so called ectopic Cushing’s syndrome, ECS) (1, 2).

Appropriate tumor localization is crucial for adequate treatment. The major limitation of imaging is that the respective tumoral lesions are usually small and therefore difficult to detect. For instance, in 30-50% of patients with CD, pituitary adenomas are initially not identified via sellar magnetic resonance imaging (MRI) (3, 4). Similarly, ectopic tumors are initially overseen in about 50% of cases (5). Furthermore, approximately 10% of the general population (6, 7) and more than 20% of patients with ECS (8) are reported to carry pituitary ‘incidentalomas’ (with the consequence of false-positive MRI results).

A thorough biochemical workup is mandatory to establish the source of ACTH hypersecretion. The baseline ACTH concentration is relatively easy to obtain and is usually remarkably higher in ECS than in CD patients (9, 10). Nevertheless, this parameter alone does not allow for a reliable differential diagnosis (10, 11). In contrast, bilateral inferior petrosal sinus sampling (BIPSS), the gold-standard for the differentiation of ACTH-dependent Cushing’s syndrome (12, 13), is a challenging and invasive procedure potentially leading to severe complications and a high radiation exposure (14, 15).

Accordingly, a step-by-step differential diagnosis is suggested (1, 16). After initial confirmation of ACTH-dependent Cushing’s syndrome, dynamic function tests like the corticotropin-releasing hormone (CRH) stimulation test and variants of the high-dose dexamethasone suppression test (DST) such as the overnight 8 mg DST are suggested to identify persistent pharmacodynamic effects that are typical for CD (i.e., stimulation of ACTH and cortisol by CRH, and suppression of cortisol by high doses of dexamethasone) (11, 17). Although both dynamic function tests are well established, some substantial discrepancies, especially regarding the cut-offs and test protocols applied, were described (9, 18–25). Furthermore, the number of reported patient with CD (ranging from 49 to 288) and ECS (ranging from 7 to 27) was limited.

The aim of this study was to evaluate the diagnostic performance of the CRH stimulation test and the overnight 8 mg DST (either alone or in combination) in a large series of patients with confirmed ACTH-dependent Cushing’s syndrome.

## Subjects and methods

### Participating centers and ethical considerations

This multicenter study was conducted in accordance with the local ethical committees of the participating centers (local ethics committee approval numbers 85/12 in Wurzburg and Berlin, NCH-01-21 in Milan, 152-10 in Munich, 353/2013BO2 in Tubingen, and 1457/2016 in Vienna).

### Subjects

Patients with ACTH-dependent Cushing’s syndrome who were diagnosed between 1984 and 2020 according to established criteria (26) were retrospectively reviewed. Those who underwent a stimulation test with administration of human CRH and/or an overnight 8 mg DST [with a single dose of 8 mg dexamethasone administered p.o. at 11.00 p.m. (27)] were considered eligible for the current evaluation. A subset of 96 patients (CD, n=78; ECS, n=18) from Munich was already published elsewhere (23).

### Standard operating procedures for the dynamic testing procedures

Only dynamic testing procedures that were performed according to standardized protocols were taken into account. CRH stimulation tests had to be carried out in the morning, with blood sampling for serum cortisol and plasma ACTH at -15 and 0 minutes, and 15, 30, 45, 60, 90, and 120 minutes after injection of 100 µg of synthetic human CRH (as shown in **Supplementary Table 1**, the distinct time points slightly differed from center to center). With respect to the overnight 8 mg DST, a baseline sample for measurement of serum cortisol was obtained between 8.00 and 9.00 a.m. Afterwards, 8 mg dexamethasone were administered as a single dose p.o. at 11.00 p.m., followed by blood sampling for serum cortisol measurement between 8.00 and 9.00 a.m. the next morning.

### Biochemical analysis

Plasma ACTH was measured by Siemens Immulite 2000 XPi (in Berlin, Tübingen, and Würzburg), Nichols Advantage ACTH assay (in Milan), DiaSorin Liaison (in Munich), and Roche Cobas (in Vienna). Serum cortisol was determined by Siemens Immulite 2000 XPi (in Berlin and Würzburg), DiaSorin Liaison (in Munich), Siemens ADVIA Centaur XPT (in Tübingen), and Roche Cobas (in Vienna). In Milan, the Tosoh Bioscience AIA-PACK CORT immunoassay was used for cortisol analysis until 2016; afterwards, the Roche Elecsys was applied.

### Interpretation of the biochemical baseline assessment and the two dynamic testing procedures

The biochemical results were interpreted as follows: a) analysis of ACTH and cortisol at baseline; b) post-CRH %-increase of ACTH and cortisol over baseline; c) post-CRH peak of ACTH and cortisol; d) post-dexamethasone %-suppression of cortisol. For this, newly generated cut-offs were applied; their diagnostic accuracy was compared to already published cut-offs for the CRH stimulation test and the overnight 8 mg DST.

### Statistical analysis

Statistical analyses were performed with SPSS version 26 (IBM Corporation, Armonk, NY, USA) and GraphPad Prism version 8 (GraphPad, San Diego, CA, USA). Data are presented as median and interquartile range (IQR). Comparisons between CD and ECS were performed with Mann-Whitney-U-test for non-normally distributed metrically scaled variables and Pearson Chi-Square for dichotomous categorical variables. For comparisons of the different study centers, Kruskal-Wallis-test for non-normally distributed metrically variables were carried out. To calculate optimal cut-offs and the associated sensitivities, specificities, and areas under the curve (AUC), receiver operator characteristic (ROC) analyses were performed, using CD as reference. In addition, the diagnostic outcome was evaluated with the Youden’s index (J = sensitivity + specificity-1).

## Results

### Clinical characteristics of the study cohort

Out of the entire retrospective cohort of 616 patients with ACTH-dependent Cushing’s syndrome (Tübingen, n=167 (27%); Munich, n=149 (24%); Vienna, n=118 (19%); Würzburg, n=108 (18%); Milan, n=47 (8%); Berlin, n=27 (4%)), 556 (90%) underwent a CRH stimulation test and/or an overnight 8 mg DST. In 469 (84%) of these patients, diagnostic confirmation was achieved either by histopathology or by the clinical outcome after surgery (i.e., biochemical remission according to common screening tests for Cushing’s syndrome and/or temporary adrenal insufficiency). Of note, only this ‘gold standard’ cohort of 469 patients was taken into account for the calculation of cut-offs and further analyses (clinical characteristics are provided in **Table 1**).

**Table 1:** Clinical characteristics of patients with histologically or post-surgically confirmed diagnosis of Cushing’s disease or ectopic Cushing’s syndrome.

	CD	ECS	p-value
**Clinical characteristics**			
Subjects [n (%)]	440 (94%)	29 (6%)	–
Females [n (%)]	348 (79%)	17 (59%)	<0.05
Age (years) [median (IQR)]	43 (19)	41 (33)	n.s.
Body mass index (kg/m²) [median (IQR)]	28 (8)	28 (8)	n.s.
Source of ACTH-dependent Cushing’s syndrome*			
Pituitary gland [n (%)]	440 (100%)	–	–
Lung [n (%)]	–	22 (76%)	–
Pancreas [n (%)]	–	4 (14%)	–
Others ** [n (%)]	–	3 (10%)	–
**Confirmatory diagnostics**			
Histology [n (%)]	359 (82%)	26 (90%)	n.s.
Post-operative remission and/or post-operative adrenal insufficiency [n (%)]	81 (18%)	3 (10%)	n.s.
**Biochemical screening tests**			
ACTH (pg/ml) *** [median (IQR)]	61 (46)	116 (111)	<0.001
Serum cortisol after 1 mg DST (nmol/l) [median (IQR)]	400 (375)	800 (761)	<0.001
24-hour urinary free cortisol (µg/d) **** [median (IQR)]	344 (454)	1634 (1906)	<0.001
Late-night salivary cortisol (nmol/l) [median (IQR)]	19 (22.0)	117 (139)	<0.001
Late-night serum cortisol (nmol/l) [median (IQR)]	477 (287)	811 (681)	<0.01

Data regarding biochemical screening tests were available from: ACTH, n = 469 (100%); 1 mg DST, n = 404 (86%); 24-hour urinary free cortisol, n = 402 (86%); late-night salivary cortisol, n = 161 (34%); late-night serum cortisol, n = 129 (28%).

ACTH, adrenocorticotropin; CD, Cushing’s disease; CRH, corticotropin-releasing hormone; DST, dexamethasone suppression test; ECS, ectopic Cushing’s syndrome; IQR, interquartile range; n.s., not significant.

*data on individual tumor grade not systematically assessed. ** each one case with a thymus carcinoma, a pheochromocytoma, and an esthesioneuroblastoma; *** collected outside the CRH stimulation test; **** despite indisputable between-center variation in biochemical analysis, 24-hour urinary free cortisol is provided for transparency.

### Basal screening parameters

As outlined in **Table 1**, all biochemical screening parameters were significantly higher in ECS than in CD patients. However, there were a remarkable overlap, and neither single screening parameters nor a combination of several screening parameters was able to differentiate well between CD and ECS (data not shown).

### CRH stimulation test

A CRH stimulation test was performed in 420 patients (CD, n=394 (94%); ECS, n=26 (6%)). Of note, the sampling time points -15 and 45 minutes were excluded from further analyses because only few samples were collected at these time points. As shown in **Supplementary Figures 1**, **2**, all six centers demonstrated a well-comparable test pattern (significant differences between the centers were only observed for ACTH at 90 minutes).

**Figure 1:**
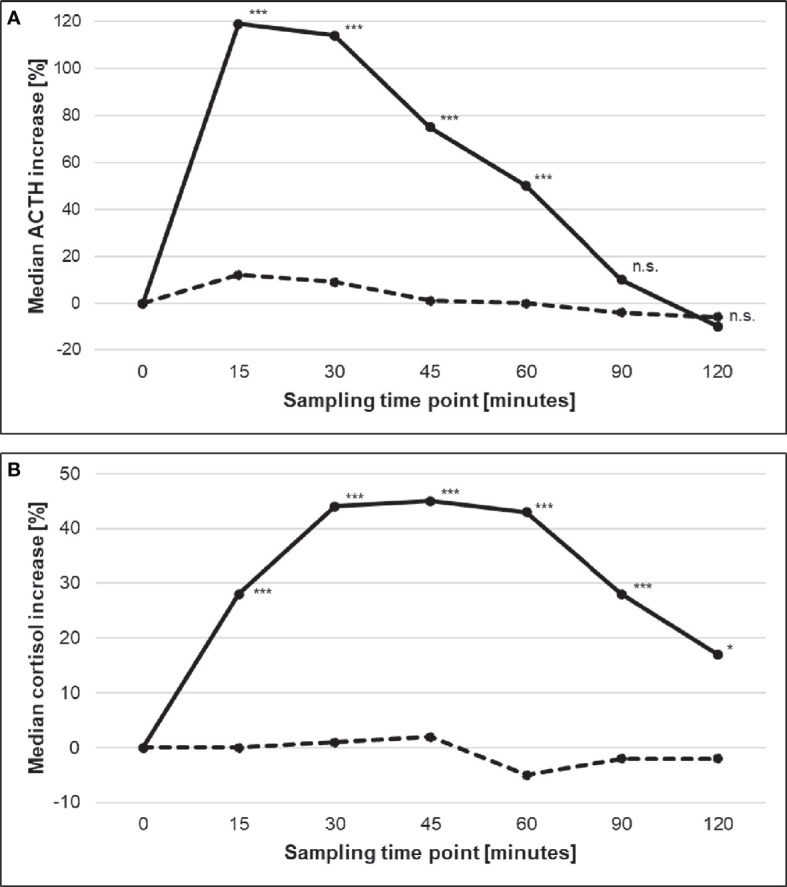
Median %-increases of **(A)** ACTH and **(B)** cortisol during the CRH stimulation test in patients with CD (solid line) and ECS (dashed line). Stars indicate statistical significant differences between both sub-entities (* <0.05; *** <0.001). ACTH, adrenocorticotropin; CD, Cushing’s disease; CRH, corticotropin-releasing hormone; ECS, ectopic Cushing’s syndrome. n.s., not significant

As shown in **Figure 1**, CD patients demonstrated substantial post-CRH responses of both ACTH and cortisol, with peak levels for ACTH at 15 minutes (median %-increase from baseline 120%, IQR 169%; **Figure 1A**) and for cortisol at 30 minutes (median %-increase from baseline 44%, IQR 55%; **Figure 1B**). In contrast, ECS patients demonstrated no relevant post-CRH changes of ACTH and cortisol. **Supplemental Table 2** provides the individual responses of ACTH and cortisol during the CRH-stimulation test for all ECS patients.

Firstly, baseline levels of ACTH and cortisol were evaluated (i.e., before CRH administration). ROC analysis revealed an optimal cut-off of 110 pg/ml for baseline ACTH (sensitivity 89%, specificity 58%; AUC 0.70) and of 883 nmol/l for baseline cortisol (sensitivity 87%, specificity 58%; AUC 0.72) (**Table 2**).

**Table 2:** Diagnostic outcome of ACTH during the CRH stimulation test.

ACTH	Time (min)	Cut-off	J	Sens. (%)	Spec. (%)	AUC	PPV (%)	NPV (%)	p-value
Baseline level (pg/ml)	0	110	0.47	89	58	0.70	97	25	–
Post-CRH %-increase	15	≥55%	0.58	73	85	0.82	99	19	<0.001
	30	≥31%	0.68	83	85	0.81	99	25	<0.001
	60	≥14%	0.56	71	85	0.76	99	18	<0.001
	90	≥14%	0.35	48	87	0.62	97	15	n.s.
	120	≥17%	0.20	29	91	0.58	97	12	n.s.
Post-CRH peak level (pg/ml)	–	160	0.15	42	73	0.56	96	8	–

ACTH, adrenocorticotropin; AUC, area under the curve; CRH, corticotropin-releasing hormone; J, Youden’s index; NPV, negative predictive value; n.s., not significant; PPV, positive predictive value; Sens., sensitivity; Spec., specificity.

**Figure 2:**
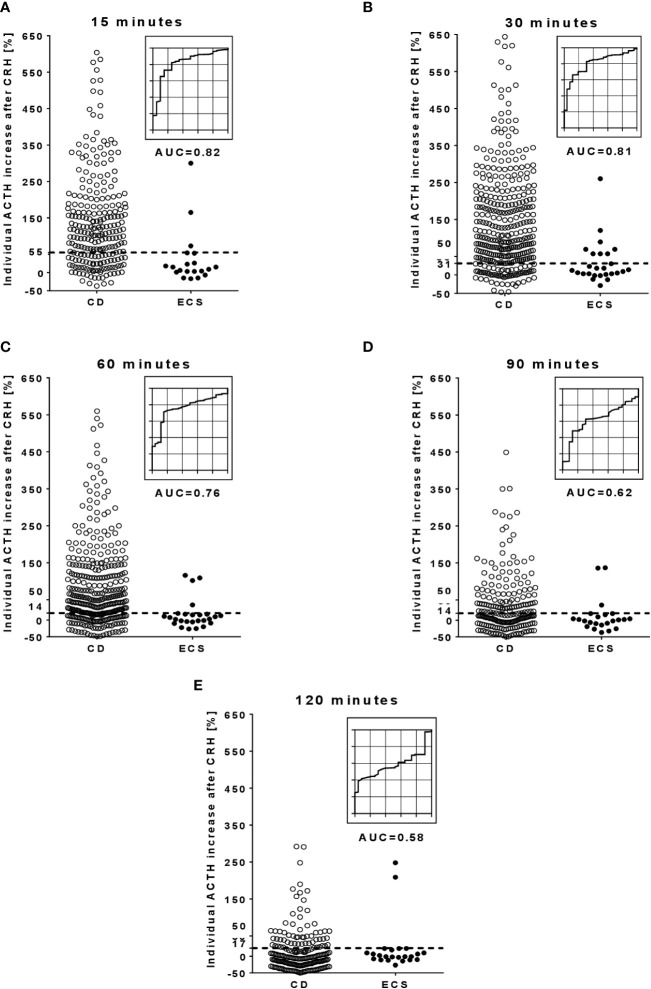
Individual %-increase of ACTH after CRH and corresponding ROC curves at different time points during the CRH stimulation test. (**A**, at 15 minutes; **B**, at 30 minutes; **C**, at 60 minutes; **D**, at 90 minutes; **E**, at 120 minutes). The dotted lines in the scatter plots illustrate the optimal cut-off for the post-CRH %-increase of ACTH. Few outlier results are not reported in the scatter plots: 13 CD patients at 15 minutes, 20 CD patients at 30 minutes, 11 CD patients at 60 minutes, and 3 CD patients at 90 minutes. ACTH, adrenocorticotropin; AUC, area under the curve; CD, Cushing’s disease; CRH, corticotropin-releasing hormone; ECS, ectopic Cushing’s syndrome; ROC, receiver operator characteristic.

**Figure 3:**
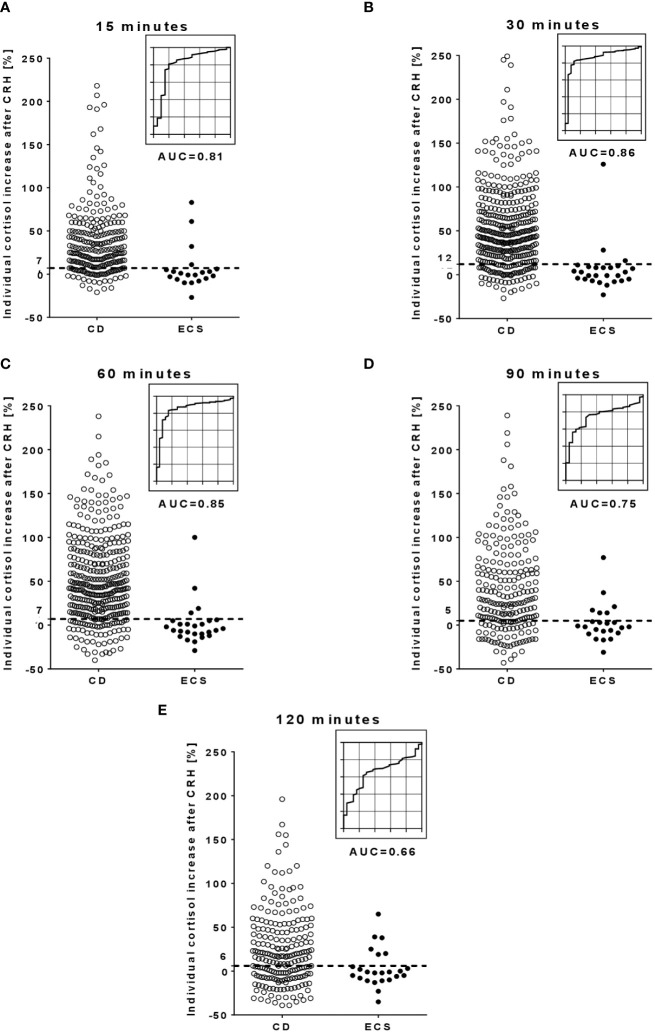
Individual %-increase of cortisol after CRH and corresponding ROC curves at different time points during the CRH stimulation test. (**A**, at 15 minutes; **B**, at 30 minutes; **C**, at 60 minutes; **D**, at 90 minutes; **E**, at 120 minutes). The dotted lines in the scatter plots illustrate the optimal cut-off for the post-CRH %-increase of cortisol. Few outlier results are not reported in the scatter plots: 1 CD patients at 15 minutes, 7 CD patients at 30 minutes, 9 CD patients at 60 minutes, 4 CD patients at 90 minutes, and 2 CD patients at 120 minutes. AUC, area under the curve; CD, Cushing’s disease; CRH, corticotropin-releasing hormone; ECS, ectopic Cushing’s syndrome; ROC, receiver operator characteristic.

Secondly, the CRH-responses of ACTH and cortisol were analyzed. **Figures 2**, **3** show the individual post-CRH %-increases of ACTH and cortisol throughout the test, along with the corresponding optimal cut-offs and ROC curves. Furthermore, **Table 2** provides the diagnostic outcome of the optimal cut-offs for the post-CRH %-increase of ACTH and cortisol. For CRH-stimulated ACTH, the cut-off with the highest Youden’s index was ≥31% at 30 minutes (sensitivity 83%, specificity 85%, AUC 0.81) (**Table 2**). The optimal cut-off for the post-CRH %-increase of cortisol was calculated as ≥12% at 30 minutes (sensitivity 82%, specificity 89%, AUC 0.86) (**Table 3**).

**Table 3:** Diagnostic outcome of cortisol during the CRH stimulation test.

Cortisol	Time (min)	Cut-off	J	Sens. (%)	Spec. (%)	AUC	PPV (%)	NPV (%)	p-value
Baseline level (nmol/l)	0	883	0.45	87	58	0.72	97	21	–
Post-CRH %-increase	15	≥7%	0.61	81	80	0.81	98	26	<0.001
	30	≥12%	0.71	82	89	0.86	99	25	<0.001
	60	≥11%	0.69	78	91	0.83	99	21	<0.001
	90	≥7%	0.68	83	85	0.85	99	26	<0.001
	120	≥5%	0.48	74	74	0.75	97	22	<0.001
Post-CRH peak level (nmol/l)	–	1048	0.22	68	54	0.57	96	10	–

AUC, area under the curve; CRH, corticotropin-releasing hormone; J, Youden’s index; NPV, negative predictive value; PPV, positive predictive value; Sens., sensitivity; Spec., specificity.

Thirdly, the diagnostic outcome of different test durations was assessed (taking the post-CRH levels of ACTH and cortisol from the 5 sampling time points 15, 30, 60, 90 and 120 minutes into account). For ACTH levels, AUC values gradually decreased from 0.82 at 15 minutes to 0.58 at 120 minutes (emphasizing a lower discriminatory power at later time points). Although less pronounced, AUC values for cortisol also decreased over time (**Tables 2**, **3**). Samples taken beyond 60 minutes allowed identification of 5 additional CD patients (2 with ACTH, 3 with cortisol) but also led to 5 false-positive ECS patients (3 with ACTH, 2 with cortisol).

Finally, the post-CRH peaks of ACTH and cortisol were analyzed. In terms of sensitivity, specificity, and AUC, however, post-CRH peaks of both parameters demonstrated a rather poor diagnostic outcome (**Tables 2**, **3**).

### Overnight 8 mg dexamethasone suppression test

**Figure 4:**
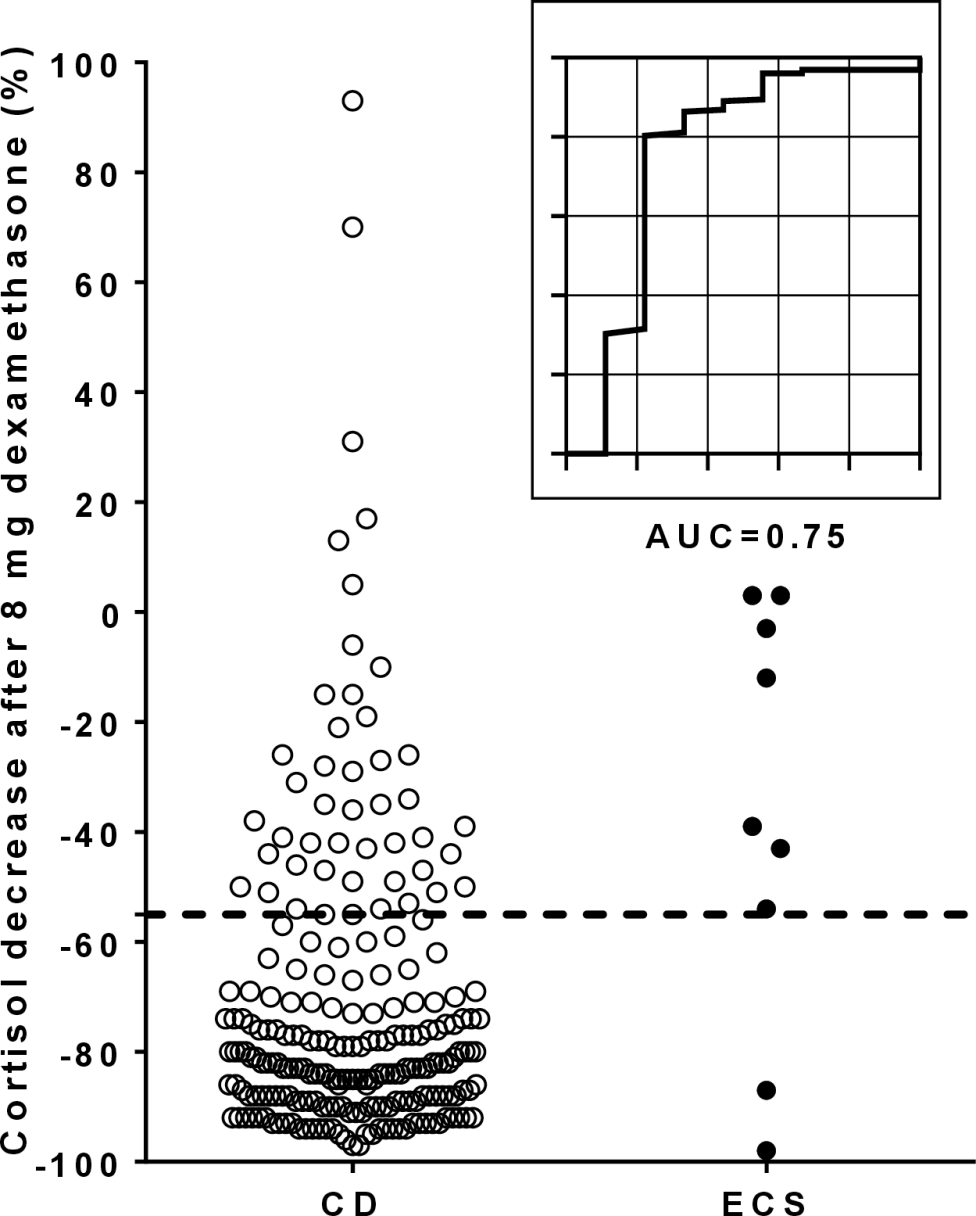
Individual %-suppression of cortisol after dexamethasone and corresponding ROC curve for the overnight 8 mg dexamethasone suppression test. The dotted line in the scatter plot illustrates the optimal cut-off of 55% for the %-suppression of cortisol after dexamethasone. One CD patient with an outlier result of 628% is not reported in the scatter plot. AUC, area under the curve; CD, Cushing’s disease; ECS, ectopic Cushing’s syndrome; ROC, receiver operating characteristic.

The overnight 8 mg DST was conducted in 237 patients (228 CD (96%), 9 ECS (4%)). The median %-decrease of cortisol after 8 mg dexamethasone was 80% (IQR 26%) in patients with CD, and 40% (IQR 71%) in patients with ECS, respectively. As illustrated in **Table 4** and **Figure 4**, ROC analysis revealed an optimal cut-off of ≥55% (sensitivity 80%, specificity 78%, AUC 0.75).

**Table 4:** Diagnostic outcome of the overnight 8 mg dexamethasone suppression test.

Cortisol	Cut-off	J	Sens. (%)	Spec. (%)	AUC	PPV (%)	NPV (%)	p-value
%-suppression	≥55%	0.58	80	78	0.75	99	14	<0.05

ACTH, adrenocorticotropin; AUC, area under the curve; overnight 8 mg DST, overnight 8 mg dexamethasone suppression test; J, Youden’s index; NPV, negative predictive value; PPV, positive predictive value; Sens., sensitivity; Spec., specificity.

The outcome of a published cut-off of ≥50% for the %-suppression of cortisol during the overnight 8 mg DST was also evaluated. In our cohort, a comparable sensitivity (83% vs. 80%) and an identical AUC (0.75) but a lower specificity (67% vs. 78%) compared to our newly calculated cut-off of ≥55% were observed.

### Combination of ACTH and cortisol during the CRH stimulation test

As outlined in **Table 5**, the combined analysis of ACTH and cortisol during the CRH stimulation test did not reveal any diagnostic benefit (as illustrated by comparable results for sensitivity, specificity, AUC, positive predictive value, and negative predictive value) compared to the analysis of any of these two parameters alone.

**Table 5:** Diagnostic outcome of a) the combined analysis of ACTH and cortisol at each time point during the CRH stimulation test and b) various combinations of the CRH stimulation test and the overnight 8 mg suppression test.

	Time	J	Sens. (%)	Spec. (%)	PPV (%)	NPV (%)
**Combined analysis of ACTH and cortisol during the CRH stimulation test**						
Optimal cut-offs for ACTH & cortisol during the CRH stimulation test*	15 min	0.47	67	80	98	17
	30 min	0.61	76	85	99	20
	60 min	0.44	67	77	98	15
	90 min	0.25	38	87	97	13
	120 min	0.04	26	78	92	10
**Combinations of CRH stimulation test and overnight 8 mg dexamethasone suppression test**						
ACTH at 30 min during the CRH stimulation test (≥31%) & overnight 8 mg DST (≥55%)		0.42	67	75	98	9
Cortisol at 30 min during the CRH stimulation test (≥12%) & overnight 8 mg DST (≥55%)		0.46	71	75	99	10
ACTH & Cortisol at 30 min during the CRH stimulation test & overnight 8 mg DST		0.39	64	75	98	9

ACTH, adrenocorticotropin; AUC, area under the curve; CRH, corticotropin-releasing hormone; overnight 8 mg DST, overnight 8 mg dexamethasone suppression test; J, Youden’s index; NPV, negative predictive value; PPV, positive predictive value; Sens., sensitivity; Spec., specificity; * the newly generated optimal cut-offs for ACTH and cortisol are provided in **Tables 2**, **3**.

### Combination of the CRH stimulation test and the overnight 8 mg dexamethasone suppression test

Both dynamic testing procedures were carried out in 205 patients (197 CD (96%), 8 ECS (4%)). Overall, various combinations of the CRH stimulation test (i.e., with ACTH only, with cortisol only, or with both ACTH and cortisol) and the overnight 8 mg DST had comparable discriminatory power to the single tests (**Table 5**). However, if at least one of the two tests (i.e., either the CRH stimulation test or the overnight 8 mg DST) indicated CD, the correct diagnosis was established in 93.0-96.0% of cases (as shown in **Supplementary Figure 3**).

## Discussion

CRH stimulation test and overnight 8 mg DST are dynamic testing procedures widely applied for the differentiation of ACTH-dependent CS (1, 9, 23, 28). In our study, we investigated the diagnostic outcome of both tests in a large number of well-characterized patients with confirmed diagnoses. We observed that ACTH and cortisol responses during the CRH stimulation test had comparable diagnostic value, and that sampling beyond 60 minutes after CRH stimulation did not provide diagnostic benefits. The overnight 8 mg DST demonstrated equivalent sensitivity but lower specificity. If both dynamic testing procedures (i.e., the CRH test with any parameter and the overnight 8 mg DST) were carried out simultaneously and any test outcome indicated CD, this was true in ≥ 93.0% of cases.

The CRH stimulation test is considered the most reliable non-invasive dynamic test in differentiating CD and ECS (9, 23, 25). Recently, high sensitivities for the non-stimulated baseline parameters were reported in a series of 101 patients with ACTH-dependent Cushing’s syndrome (87% for ACTH vs. 93% for cortisol) (29). Although we observed comparable sensitivities (89% for ACTH vs. 87% for cortisol), specificity was remarkably lower (each 58% in our study vs. reported data of 69% for ACTH and 93% for cortisol). Accordingly, we have the impression that additional CRH stimulation appears justified.

In our series, the optimal cut-offs for the post-CRH %-increase at 30 minutes (≥31% for ACTH and ≥12% for cortisol) demonstrated comparable sensitivity (83% vs. 82%) and only moderate differences in specificity (85% vs. 89%).

Compared to the literature, however, the post-CRH %-increases of ACTH that were observed in our study had remarkably lower specificities despite similar sensitivities (9, 22, 23, 28). A possible explanation is certainly the limited number of ECS patients in other studies, making false-positive results per se less likely. In fact, it is well known that some neuroendocrine tumors and bronchial carcinoids (despite excessively high ACTH levels) still respond to a CRH stimulus (30), and this was also true for 8 (31%) of our ECS patients. In particular, four ECS cases with low baseline levels of ACTH and cortisol showed a remarkable post-CRH increase of both parameters, what is possibly related to a diminished negative feedback inhibition of the hypothalamus-pituitary-adrenal axis (which is considered to be a typical feature in ECS) (23, 31). On the contrary, four other ECS cases had incongruent results (post-CRH increase only of cortisol, n=3; post-CRH increase only of ACTH, n=1), most likely reflecting false-positive results (e.g. due to multiple sampling time points, as a tendency towards higher ACTH and cortisol levels was observed over time).

A pertinent finding of our study is the diagnostic value of cortisol analysis during the CRH stimulation test, a result that is different to a former manuscript on a subgroup of our current study cohort (23). Nevertheless, our current findings have also been reported by others (9, 18, 22, 24, 32). In two studies involving stimulation with human CRH (as in our study), post-CRH cortisol cut-offs of ≥14% (9) and ≥17% (24) resulted in sensitivities of 85% and 90%, and specificities of 100% and 85%, respectively. Although both reported cut-offs are well comparable to our current cut-off of ≥12%, discrepancies regarding sensitivity and specificity may possibly be explained by a) the remarkably lower number of CD patients in former publications (i.e., 101 and 167 in former vs. 420 in this series) and b) the different study outlines (e.g. overall instead of time-point specific analysis of the %-increase) (24). Regarding the latter point, for instance, the trend in ECS patients towards higher ACTH and cortisol levels over time (that was already mentioned above) may result in more false-positive results if overall instead of time-point specific cut-offs are applied.

With respect to other studies (20, 24, 31), contradictory results regarding the analytical merits of cortisol during the CRH-stimulation test may also be explained by the use of ovine CRH instead of human CRH. According to some authors, ovine CRH results in a prolonged and more pronounced response of both ACTH and cortisol due to a longer plasma half-life and a lower metabolic clearance rate (22, 33). On the other hand, other studies reported comparable effects of human and ovine CRH (20, 21). A direct comparison between the two compounds would certainly be of interest, however, their commercial availability is limited (oCRH is not available in Europe and United States, and hCRH is not available in the United States).

Considering that the maximal discriminatory power of post-CRH %-increase of ACTH and cortisol was achieved at 30 minutes, it is our impression that a duration of the test beyond 60 minutes does not appear to be useful. This confirms what was already reported elsewhere (9, 23, 34).

The high-dose DST represents an alternative to the CRH stimulation test in the in the differentiation of CD and ECS (28). Several protocols are known, but the overnight 8 mg DST represents one of the most widely applied variants. Confirming what has been already reported by others (19, 23), we observed that this test demonstrated lower diagnostic accuracy than the CRH stimulation test (hence, this procedure is also not recommended in a current consensus paper (16)). With respect to our study, application of the newly generated optimal cut-offs led to similar sensitivities (overnight 8 mg DST: 80%; CRH stimulation test: 83% for ACTH, 82% for cortisol), however, specificity was lower (overnight 8 mg DST: 78%; CRH stimulation test: 85% for ACTH, 89% for cortisol). A possible explanation might be the persistent dexamethasone responsiveness of some neuroendocrine tumors (30, 35). Of note, the low number of our ECS patients undergoing an overnight 8 mg DST (n=9) certainly represents a relevant limitation of our current analysis.

Our suggested optimal cut-off of ≥55% for dexamethasone suppressed serum cortisol was well comparable to a published threshold of ≥53% (19). Both cut-offs, however, demonstrated a remarkably discrepant diagnostic outcome, as illustrated by sensitivities of 80% and 88%, and specificities of 78% and 90%, respectively (with the lower values observed in our own study). However, if we applied the conventional cut-off of ≥50%, we identified a comparable sensitivity (of 83%) and specificity (of 67%) to what has been previously reported elsewhere (i.e., a sensitivity of 81%, and a specificity of 67%) (19).

Interestingly, if the CRH stimulation test and the overnight 8 mg DST were analyzed in combination, sensitivity and specificity decreased substantially (to 64-71% and to 75%, depending on the particular combination), while the positive predictive value remained remarkably high (always ≥98%). In two other studies, higher sensitivities (of 76% and 81%) and specificities (of 89% and 100%) were reported (20, 36). This discrepancy could probably (at least in part) be explained by the highly variable numbers of patients with CD (ranging from 148 to 420) and ECS (ranging from 8 to 26) in the threes studies. Nevertheless, each single test obviously allowed for a better diagnostic outcome. Accordingly, one could argue that a diagnostic routine approach (with both testing procedures being carried out in each individual) appears questionable. Recently, however, it was shown that a concordant positive result to both dynamic tests may be sufficient to reliably diagnose CD in patients with negative MRI but subsequently confirmed small pituitary microadenomas (36). Furthermore, if both dynamic testing procedures were applied simultaneously and at least one test indicated CD, we observed that this finding was true in ≥93.0% of our cases. In other words, the vast majority of patients with CD who undergo pituitary surgery on the basis of such test combinations will be adequately treated.

Due to its retrospective and multicentric nature, our current study has certainly some important limitations (e.g. center-specific laboratory testing procedures, few individuals with both tests, some individuals with relatively low basal ACTH levels despite confirmed ACTH-dependent Cushing’s syndrome). However, we have the impression that these aspects reliably reflect real-world settings. Furthermore, although some authors reported assay-specific spurious ACTH levels leading to diagnostic and therapeutic obstacles (37), the center-specific analytical methodology virtually remained the same over time (in particular, only one center changed its cortisol assay). One of the most relevant boundaries is probably the low number of ECS cases that were also not comparably distributed among the six study centers. Possible gender-specific differences in test outcomes could therefore not be evaluated (as only 17 females and 12 males with ECS were enrolled). The following facts are also relevant limitations: a) data on tumor grade was not systematically assessed (as a substantially variable secretion pattern of ACTH and probably also of CRH in high- and low-differentiated tumors has to be assumed); b) radiological procedures relevantly improved over time (possibly, some of our older ECS cases had a false-negative imaging); c) only a single baseline value before administration of hCRH was analyzed (and not a mean from the two time points -15 minutes and 0 minutes). Finally, it has to be pointed out that percent increases and their respective cut-offs always have to be interpreted with caution (and should be reserved for patients with baseline levels of ACTH and cortisol in a suspiciously elevated range).

In conclusion, ACTH and cortisol measurement 30 minutes after CRH stimulation showed a comparable diagnostic outcome. The overnight 8 mg DST has significantly lower specificity than the CRH stimulation test. Finally, a duration of more than 60 minutes for the CRH stimulation test does not provide substantial diagnostic benefits. Further diagnostic procedures (e.g. BIPSS) may be omitted in cases where both dynamic tests indicate CD, however, the final decision on the required means has to be made on an individual basis.

## Data availability statement

The original contributions presented in the study are included in the article/**Supplementary Material**. Further inquiries can be directed to the corresponding author.

## Ethics statement

The studies involving human participants were reviewed and approved by local ethics committee approval numbers 85/12 in Wurzburg and Berlin, NCH-01-21 in Milan, 152-10 in Munich, 353/2013BO2 in Tubingen, and 1457/2016 in Vienna). All patients provided written informed consent.

## Author contributions

TD designed the research. MD, VT, and TD performed the statistical analyses and drafted the manuscript. All authors collected samples and clinical data from patients, contributed to writing the manuscript, and approved the final version to be published.

## Funding

This work was supported by the DFG German Research Foundation Project 314061271-TRR 205 (to MK and MF) and the European Reference Network on Rare Endocrine Conditions (Endo-ERN). This publication was supported by the Open Access Publication Fund of the University of Würzburg.

## Acknowledgments

We would like to thank Yvonne Möhres and Stephanie Zopp for data collection.

## Conflict of interest

The authors declare that the research was conducted in the absence of any commercial or financial relationships that could be construed as a potential conflict of interest.

## Publisher’s note

All claims expressed in this article are solely those of the authors and do not necessarily represent those of their affiliated organizations, or those of the publisher, the editors and the reviewers. Any product that may be evaluated in this article, or claim that may be made by its manufacturer, is not guaranteed or endorsed by the publisher.

## Supplementary material

Supplementary Figure 1 Center-specific outcome of ACTH analysis during the CRH stimulation test.

Supplementary Figure 2 Center-specific outcome of cortisol analysis during the CRH stimulation test.

Supplementary Figure 3 Diagnostic outcome of the combined analysis of the CRH stimulation test (with analysis of ACTH only, with analysis of cortisol only, and with analysis of both ACTH and cortisol) and the overnight 8 mg dexamethasone suppression test. The tables at the bottom of the graph provide details on incongruent test results. A ‘+’ indicates a true positive test result (i.e., the ACTH source was correctly identified according to the pre-defined ‘gold standard’ criteria), whereas a ‘-’ indicates a false negative test result (i.e., the ACTH source was falsely calssified according to the pre-defined ‘gold standard’ criteria).

Supplementary Table 1 Center-specific number of patients undergoing a CRH stimulation test.

Supplementary Table 2 Individual outcome of the CRH stimulation test in patients with ectopic Cushing´s syndrome.
