# Supplementary figures and images for: Outcome of CRH stimulation test and overnight 8 mg dexamethasone suppression test in 469 patients with ACTH-dependent Cushing’s syndrome
Source: Front Endocrinol (Lausanne). 2022 Oct 6;13:955945. doi: 10.3389/fendo.2022.955945 (PMC9583401; doi:10.3389/fendo.2022.955945)

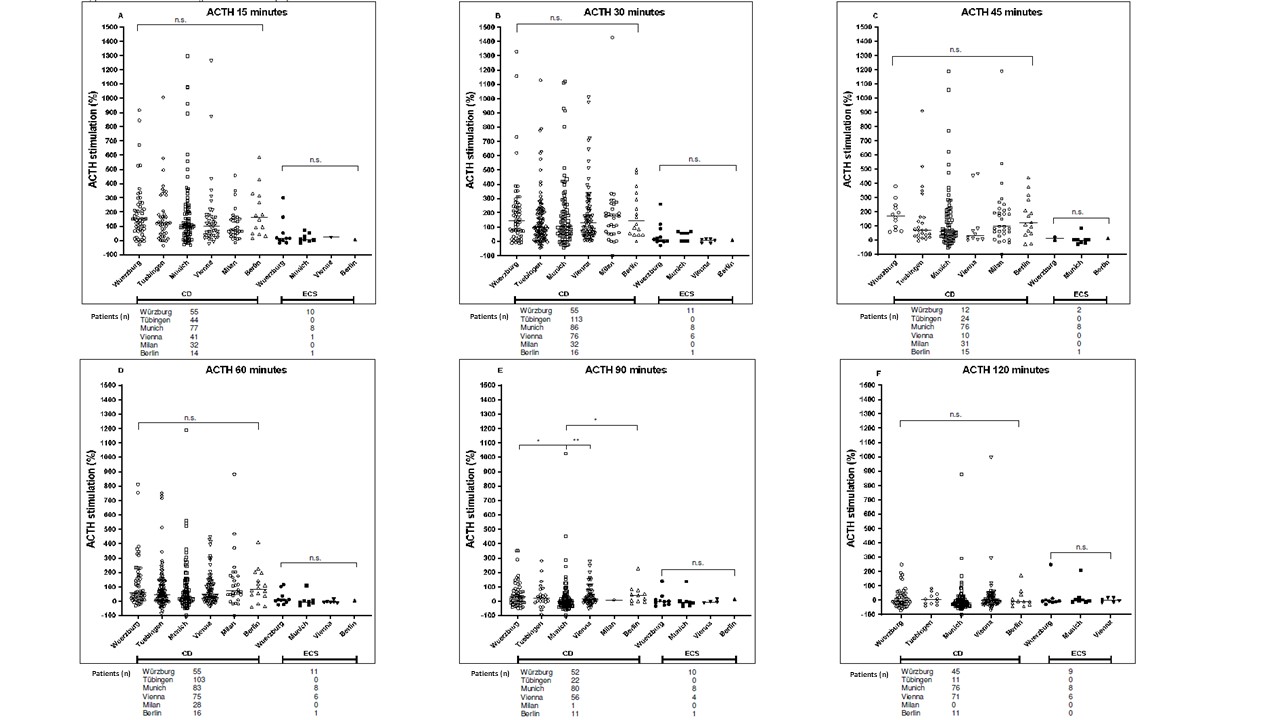

Supplement: Supplementary Figure 1 — Center-specific outcome of ACTH analysis during the CRH stimulation test. [file Image_1.jpg]

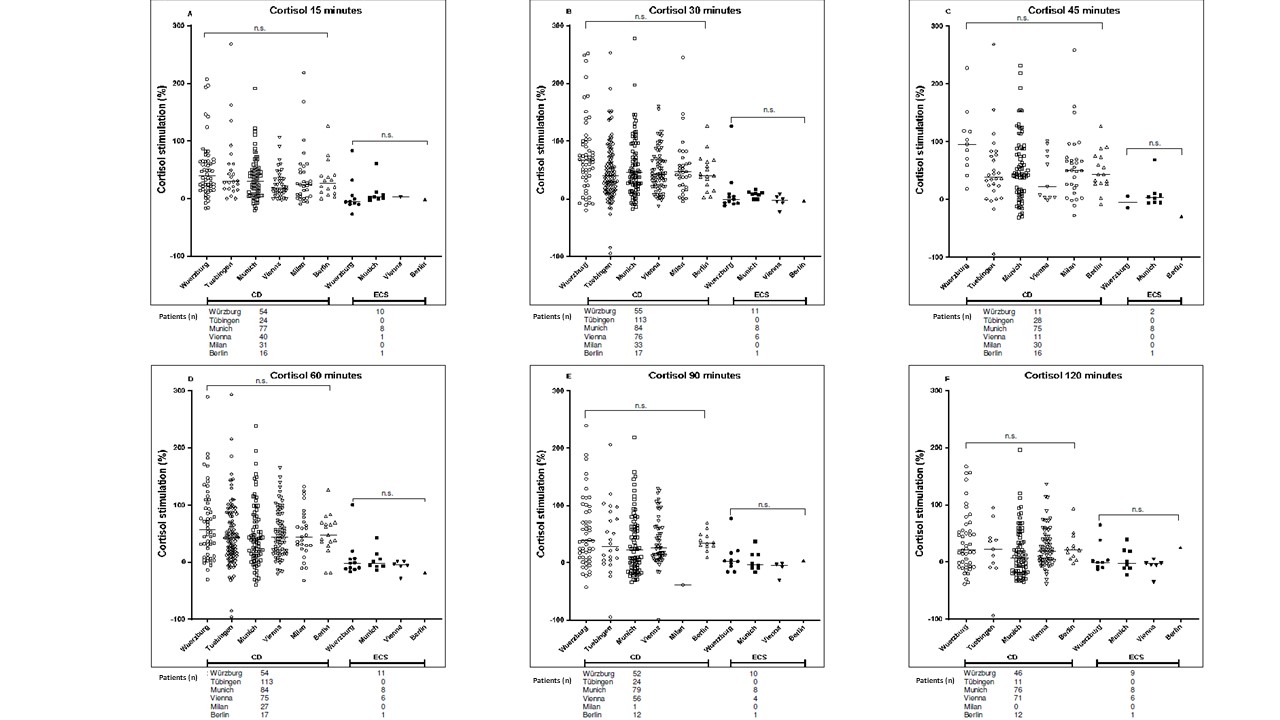

Supplement: Supplementary Figure 2 — Center-specific outcome of cortisol analysis during the CRH stimulation test. [file Image_2.jpg]

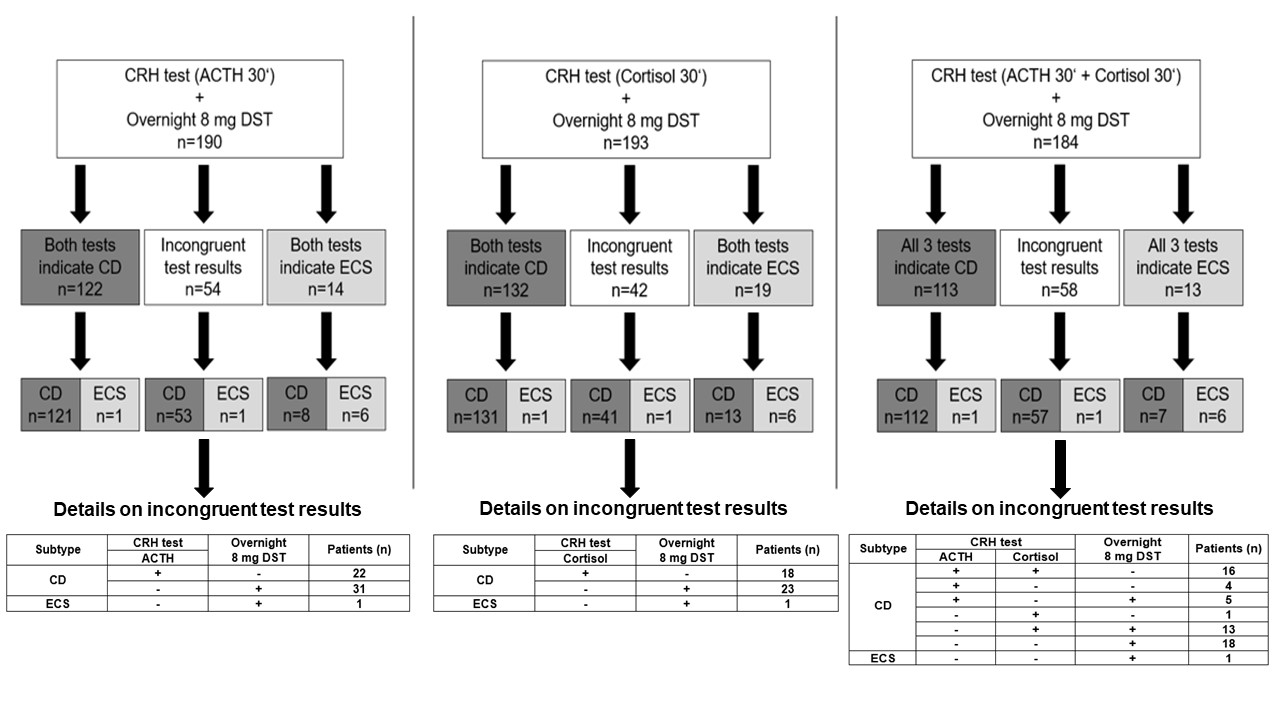

Supplement: Supplementary Figure 3 — Diagnostic outcome of the combined analysis of the CRH stimulation test (with analysis of ACTH only, with analysis of cortisol only, and with analysis of both ACTH and cortisol) and the overnight 8 mg dexamethasone suppression test. The tables at the bottom of the graph provide details on incongruent test results. A ‘+’ indicates a true positive test result (i.e., the ACTH source was correctly identified according to the pre-defined ‘gold standard’ criteria), whereas a ‘-’ indicates a false negative test result (i.e., the ACTH source was falsely calssified according to the pre-defined ‘gold standard’ criteria). [file Image_3.jpg]
