# Supplementary material for: Outcome of CRH stimulation test and overnight 8 mg dexamethasone suppression test in 469 patients with ACTH-dependent Cushing’s syndrome
Source: Front Endocrinol (Lausanne). 2022 Oct 6;13:955945. doi: 10.3389/fendo.2022.955945 (PMC9583401; doi:10.3389/fendo.2022.955945)
Supplement: Supplementary Table 1 — Center-specific number of patients undergoing a CRH stimulation test. [file Table_1.pdf]

Supplementary Table 1. Center-specific number of patients undergoing a CRH stimulation test.

Cushing subtype	Time point (min)	All patients (n)	Berlin (n)	Milan (n)	Munich (n)	Tübingen (n)	Vienna (n)	Würzburg (n)
**ACTH analysis after CRH stimulation**								
CD	15	263	14	32	77	44	41	55
	30	378	16	32	86	113	76	55
	45	168	15	31	76	24	10	12
	60	360	16	28	83	103	75	55
	90	222	11	1	80	22	56	52
	120	214	11	0	76	11	71	45
ECS	15	20	1	0	8	0	1	10
	30	26	1	0	8	0	6	11
	45	11	1	0	8	0	0	2
	60	26	1	0	8	0	6	11
	90	23	1	0	8	0	4	10
	120	23	0	0	8	0	6	9
**Cortisol analysis after CRH stimulation**								
CD	15	242	16	31	77	24	40	54
	30	378	17	33	84	113	76	55
	45	171	16	30	75	28	11	11
	60	370	17	27	84	113	75	54
	90	224	12	1	79	24	56	52
	120	216	12	0	76	11	71	46
ECS	15	20	1	0	8	0	1	10
	30	26	1	0	8	0	6	11
	45	11	1	0	8	0	0	2
	60	26	1	0	8	0	6	11
	90	23	1	0	8	0	4	10
	120	24	1	0	8	0	6	9

Abbreviations: ACTH, adrenocorticotropin; CD, Cushing's disease; CRH, corticotropin releasing hormone; ECS, ectopic Cushing's syndrome; min, minutes; n, number.
