# Supplementary material for: Outcome of CRH stimulation test and overnight 8 mg dexamethasone suppression test in 469 patients with ACTH-dependent Cushing’s syndrome
Source: Front Endocrinol (Lausanne). 2022 Oct 6;13:955945. doi: 10.3389/fendo.2022.955945 (PMC9583401; doi:10.3389/fendo.2022.955945)
Supplement: Supplementary Table 2 — Individual outcome of the CRH stimulation test in patients with ectopic Cushing´s syndrome. [file Table_2.pdf]

Supplementary Table 2. Individual outcome of the CRH stimulation test in patients with ectopic Cushing´s syndrome.

Patient	Localization of the tumor	ACTH (pg/ml)						Cortisol (nmol/l)					
		0 min	15 min	30 min	60 min	90 min	120 min	0 min	15 min	30 min	60 min	90 min	120 min
1	Adrenal	13	50	45	27	17	12	336	615	761	673	595	463
2	Pancreas	17	21	32	35	41	60	521	491	518	515	629	858
3	Lung	115	132	116	125	119	106	1686	1547	1732	1782	1732	1729
4	Lung	48	-	55	66	-	-	552	-	524	496	-	-
5	Pancreas	11	28	23	12	8	9	491	645	626	582	573	499
6	Lung	177	147	173	166	158	167	2842	2074	2621	2361	2389	2461
7	Lung	81	83	85	86	78	82	579	524	527	510	546	571
8	Lung	159	136	113	122	124	135	1346	1280	1186	1216	1368	1335
9	Lung	146	223	190	110	96	104	1164	1051	1087	1012	973	1045
10	Lung	78	89	97	86	86	87	830	825	860	816	824	767
11	Lung	123	124	120	125	122	-	1070	1125	1156	1122	1114	-
12	Lung	81	88	93	79	78	76	775	819	849	722	733	737
13	Lung	11	19	27	23	26	34	306	493	355	435	419	424
14	Pancreas	294	306	317	253	242	252	2185	2171	2342	2044	1967	1950
15	Lung	44	41	43	41	41	51	571	634	620	648	653	678
16	Lung	78	120	60	56	48	73	1079	1048	1067	987	982	957
17	Lung	114	144	131	124	98	115	996	1023	1109	1004	976	996
18	Pancreas	212	218	220	220	209	221	1327	1351	1437	1396	1509	1591
19	Lung	34	29	25	24	24	29	190	193	187	162	157	146
20	Lung	111	-	118	103	97	108	905	-	894	866	841	885
21	Lung	193	238	216	187	188	201	1286	1329	1318	1296	1241	1263
22	Lung	135	-	159	123	-	111	996	-	1076	1007	-	943
23	Lung	135	-	118	121	125	113	1895	-	1465	1343	1307	1238
24	Lung	114	-	134	129	129	132	698	-	665	653	687	656
25	Lung	438	-	385	382	-	507	1429	-	1332	1321	-	1500
26	Lung	182	193	199	191	206	-	1451	1415	1396	1175	1489	1807

Abbreviations: ACTH, adrenocorticotropin; CRH, corticotropin releasing hormone; min, minutes.
